# On the evolutionary origins of equity

**DOI:** 10.1371/journal.pone.0173636

**Published:** 2017-03-21

**Authors:** Stéphane Debove, Nicolas Baumard, Jean-Baptiste André

**Affiliations:** 1 Institut de Biologie de l’Ecole normale supérieure (IBENS), INSERM 1024, CNRS 8197, Ecole normale supérieure - PSL Research University, Paris, France; 2 Institut Jean-Nicod (CNRS - EHESS - ENS), Département d’Etudes Cognitives, Ecole normale supérieure - PSL Research University, Paris, France; 3 Institut des Sciences de l’Évolution, UMR 5554 - CNRS – Université Montpellier 2, Montpellier, France; Tianjin University of Technology, CHINA

## Abstract

Equity, defined as reward according to contribution, is considered a central aspect of human fairness in both philosophical debates and scientific research. Despite large amounts of research on the evolutionary origins of fairness, the evolutionary rationale behind equity is still unknown. Here, we investigate how equity can be understood in the context of the cooperative environment in which humans evolved. We model a population of individuals who cooperate to produce and divide a resource, and choose their cooperative partners based on how they are willing to divide the resource. Agent-based simulations, an analytical model, and extended simulations using neural networks provide converging evidence that equity is the best evolutionary strategy in such an environment: individuals maximize their fitness by dividing benefits in proportion to their own and their partners’ relative contribution. The need to be chosen as a cooperative partner thus creates a selection pressure strong enough to explain the evolution of preferences for equity. We discuss the limitations of our model, the discrepancies between its predictions and empirical data, and how interindividual and intercultural variability fit within this framework.

## Introduction

For centuries, philosophers have emphasized the important role of proportionality in human fairness. In the fourth century BC, Aristotle suggested an “equity formula” for fair distributions [1], mathematical equivalent of “reward according to contribution,” whereby the ratios between the outputs O and inputs I of two persons A and B are made equal: $\frac{O_{A}}{I_{A}}=\frac{O_{B}}{I_{B}}$. This formula also captures the concept of “merit,” the idea that people who work harder deserve more benefits [2–4].

Psychological research on distributive justice, and on equity theory in particular, has offered extensive empirical support for Aristotle’s claim [2, 5–7]. Equity theory aims to predict the situations in which people will find that they are treated unfairly. A robust finding is that receiving more or less than what one deserves leads to distress and attempts to restore equity by increasing or decreasing one’s contribution [2, 8]. People prefer income distributions with strong work-salary correlations, prefer to give more to individuals whose input is more valuable, and favor meritocratic distributions as a whole in both micro- and macro-justice contexts [9].

More recently, experiments with economics games have shown that participants consistently divide the product of cooperative interactions in proportion to each individual’s talent, effort, and the resources invested in the interaction [10, 11]. Meritocratic distributions have been observed across many societies [12], including hunter-gatherer societies [13–16], and can be detected very early in human development [17, 18], suggesting that equity could be a universal and innate pattern in human psychology.

Preferences for equitable outcomes present the same evolutionary problem as preferences for fair outcomes in general: at least in the short term, those preferences are costly. Although people react more to inequitable situations when they are disadvantageous than when they are advantageous, people still feel uncomfortable in unjustified advantageous situations [19, 20]. Experiments even show that people are ready to incur costs and decrease their own payoff in order to achieve more equitable distributions [21]. How can natural selection account for the evolution of such costly preferences?

Until now, little attention has been given to this question. There have been many theoretical studies on the evolution of fairness [22–27], but all of them are concerned with explaining the evolution of fairness in the ultimatum game, an economic game where the fair division happens to be a division into two equal halves [28, 29]. However, equal divisions are just a special case of the more general category of equitable divisions: that is, divisions proportional to contributions. As emphasized by equity theory, unequal divisions can be judged fair when they respect the partners’ investment, talents, commitment, etc. In brief, although many models can explain the evolution of preferences for *equal* divisions, none of them is able to explain the evolution of preferences for *proportional* divisions. Here we aim to understand whether natural selection can lead to such proportional divisions of resources (including the particular case of equal divisions), in a scenario where partners can make differing contributions to a cooperative undertaking.

Partner choice has had an important role in the evolution of cooperation, as evidenced by both theoretical [30–34] and empirical studies ([35–37], and see [38] for a review in humans). When people are in competition to be chosen as cooperative partners, experiments show that they increase their level of cooperation because they have a direct interest in doing so [35, 39]. Partner choice also has interesting consequences for the evolution of fairness. It leads to equal divisions of resources in theoretical and empirical settings [26, 27, 40], because when individuals can choose whom to cooperate with then they are better off refusing divisions that do not compensate their opportunity costs. These results suggest the way through which partner choice could also explain the evolution of divisions proportional to contributions: if greater contributors have larger opportunity costs, they will choose partners who give them something at least equal to these opportunity costs. Nonetheless, this hypothesis has never been studied formally (with the exception of [41], published at the same time as this article).

To summarize, preferences for equity are robust and widespread in humans, but we currently lack an evolutionary explanation for their costly existence. Here, we aim to put the partner choice mechanism to the test to see if it can explain such preferences. We develop models in which individuals put effort into the production of a collective good, and differ with regard to both the amount of effort they are willing to put in and the efficiency of their contribution to the production of the good. To determine the evolutionarily stable sharing strategy in this environment, we first analyzed an evolutionary model using agent-based simulations. We then developed a simple analytical model to better understand the simulations, and tested the robustness of our results by performing simulations with evolving neural networks as more realistic decision-making devices. The results provide converging support for the conclusion that when individuals can choose whom to cooperate with, equity emerges as the best strategy, and the offers that maximize fitness are those that are proportional to the individual’s relative contribution to the production of the good.

## Methods

We develop three complementary sets of simulations and an analytical model. For clarity, we present the first set of simulations in details before explaining how the other sets differ. Source code for all simulations is available online.

### Simulations set 1: Two productivities

#### Individuals

We consider a population of *n* individuals who will be given multiple opportunities to cooperate and produce resources during their life. Cooperation only takes place in dyadic interactions. We assume individuals are characterized by a “productivity”, such that some individuals can produce more resources than others when they cooperate. Individuals can be of one of two productivities: low-productivity individuals can produce *a* resources when they cooperate, while high-productivity individuals can produce *b* resources (*b* > *a*). This productivity is constant across the entire life of an individual but is not heritable: at birth, each individual is randomly attributed a level of productivity that is independent of his parent’s. This condition is necessary so that there is always a diversity of productivities in the population at each generation.

To decide with whom they will cooperate and how to divide resources, we assume that each individual is characterized by eight genetic variables: four *r*_*ij*_ and four MAR_*ij*_ variables, with *i* and *j* ∈ {*HP*, *LP*}, denoting an individual’s productivity (HP = High-Productivity, LP = Low-Productivity). *r*_*ij*_ is the fraction of resources (between 0 and 1) that an individual of productivity *i* will give to an individual of productivity *j*. We call the *r*_*ij*_ variables the “reward” variables. MAR_*ij*_ is the minimum acceptable reward, the minimum fraction of resource that an individual of productivity *i* is ready to accept from an individual of productivity *j*.

#### Social life

Only two types of events can happen at any given time in our model: the encounter of two solitary individuals, or the split of two cooperating individuals. We model time continuously. At each loop of the model, we (i) determine the time period until the next event (ii) determine whether this event is an encounter or a split, and (iii) execute the corresponding actions for each event, described below. This process is repeated until time has exceeded a constant *L*, which corresponds to the end of the life of all individuals (see section “reproduction” below).

After any event occurring at time *t* (or after the birth of individuals at t = 0), the time period until the next event is drawn in an exponential distribution of parameter

$$\begin{array}{c} \lambda \left(t\right)=(C\left(t\right)*\frac{\tau }{2})+S\left(t\right)*\beta \end{array}$$ (1)

with *C*(*t*) the number of cooperating individuals at time *t*, *S*(*t*) the number of solitary individuals at time *t*, *β* a constant encounter rate and *τ* a constant split rate.

The probability *p*(*t*) that this event is an encounter is then given by

$$\begin{array}{c} p\left(t\right)=S\left(t\right)*\frac{\beta }{\lambda (t)} \end{array}$$ (2)

Conversely, 1 − *p*(*t*) is the probability that this event is a split.

Depending on whether the event is an encounter or a split, two scenarios unfold:

1/ If the event is an encounter, two solitary individuals are randomly drawn from the population and offered an opportunity to cooperate to produce resources. To this end, one of the two individuals is randomly selected to unilaterally decide how to divide the resources through her *r*_*ij*_ reward variable. We call this individual the “partner”. However, before cooperation effectively starts, the partner must be accepted by the second individual. We call the second individual the “decision maker”. The decision maker makes her decision based on her partner’s reputation. For simplicity, we do not model the formation of this reputation. We simply assume that the decision maker knows her partner’s reward value *r*_*ij*_. For instance, a HP partner *A* has a reputation of *r*_*A*_*HPLP*__ with a LP decision maker *B*. The LP decision maker will then compare the value of *r*_*A*_*HPLP*__ to her own MAR_*B*_*LPHP*__, and if *r*_*A*_*HPLP*__ ≥ MAR_*B*_*LPHP*__, the partner will be accepted and cooperation will start. From this point on until the interaction stops, the two individuals produce, at each unit of time, an amount of resources that is equal to the sum of their respective productivities, from which the decision maker receives a fraction *r*_*A*_*HPLP*__. Conversely, if the partner’s reputation is not good enough for the decision maker (*r*_*A*_*HPLP*__ < MAR_*B*_*LPHP*__), the two individuals do not cooperate together and go back to the pool of solitary individuals without receiving any resources.

2/ If the event is a split, a pair of cooperating individuals is randomly chosen to split, and the two individuals go back to the pool of solitary individuals.

#### The cost of partner choice

The cost of partner choice is implicit in our model. It is a consequence of the time it takes to find a partner. Hence, the cost and benefit of being choosy are not controlled by explicit parameters, but by two parameters that characterize the “fluidity” of the social market: the “encounter rate” *β*, and the “split rate” *τ*. When $\frac{\beta }{\tau }$ is large, interactions last a long time (low split rate *τ*) but finding a novel partner is fast (high encounter rate *β*), and individuals thus should be picky about which partners they accept. This is a situation where partner choice is not costly. On the contrary, when $\frac{\beta }{\tau }$ is low, interactions are brief but finding a novel partner takes time, and individuals should thus accept almost any partner. Partner choice is then costly.

#### Reproduction

We model a Wright-Fisher population with non-overlapping generations: when the lifespan *L* has been reached, all individuals reproduce and die at the same time. The number of offsprings produced by a focal individual is given by:

$$\begin{array}{c} \text{offsprings}=\text{round}(\frac{f*z}{\bar{z}}) \end{array}$$ (3)

with *z* the focal individual’s amount of resources accumulated throughout her life, $\bar{z}$ the average amount of resources accumulated in the population, and *f* a constant multiplication factor. Offsprings receive the four *r*_*ij*_ and four MAR_*ij*_ traits from their parents, with a probablity *m* of mutation on each trait. Mutations are drawn from a normal distribution centered around the trait value with standard deviation *d*, and constrained in the interval [0, 1]. After mutations take place, *n* individuals are randomly drawn from the pool of offsprings to constitute the population for the next generation.

Table 1 summarizes the model’s parameters. To obtain the results presented below, we initialize all simulations with a population of stingy and undemanding individuals, who do not share when they play the role of partner and accept any partner when they play the role of decision maker (*r*_*ij*_ = 0, *MAR*_*ij*_ = 0). We then test our hypothesis that partner choice can lead to equitable divisions by observing how rewards and MARs evolve across generations, in two conditions: when partner choice is costly (low $\frac{\beta }{\tau }$), and when partner choice is not costly (large $\frac{\beta }{\tau }$). In particular, we will observe the rewards given by LP individuals to HP individuals at the equilibrium when partner choice is not costly, to detect whether they show the same pattern of proportionality between contribution and reward than the one observed in the empirical human data.

**Table 1:** Parameters of the model, and values used to obtain the figures presented in the main text. Deviations from these values do not change the core results.

Parameter name	Description	Value used to obtain reported results
n	number of individuals	500
a	productivity of low-productivity individuals	1
b	productivity of high-productivity individuals	2
r	reward, fraction of resources that an individual agrees to give to another	evolving (starts at 0)
MAR	minimum accepted reward, minimum fraction of resource that an individual is ready to accept	evolving (starts at 0)
*β*	encounter rate	from 0.0001 to 1
*τ*	split rate	0.01
L	lifespan	500
m	mutation rate	0.002
d	mutation standard deviation	0.02

### Analytical model

We develop an analytical model that incorporates all of the features of the simulations presented above, but with one simplification: we assume that the total number of interactions accepted per unit of time is the same for each individual. With this assumption, rejecting an opportunity to cooperate does not compromise the chances of cooperating later, but on the contrary grants new opportunities. This situation is analogous to the condition where $\frac{\beta }{\tau }$ tends towards infinity in the simulations: social opportunities are plentiful at the scale of the length of interactions. The analysis of this model is presented in details in SI section 2.

### Simulations set 2: A continuum of productivities

Introducing a continuum of productivities is necessary to get closer to biological reality. Rather than having only two productivities *a* and *b* in our population, we assume in Simulations Set 2 that the productivity of an individual at birth is sampled from a uniform distribution between *a* and *b*. In this situation, individuals never interact with a partner of the exact same productivity. This constitutes a challenge for modeling in that individuals would need to be equipped with an infinity of *r*_*ij*_ and MAR_*ij*_ traits to react to the infinity of possible contributions by their partner [42].

To solve this problem, we do not characterize anymore individuals with *r*_*ij*_ and MAR_*ij*_ traits, but instead endow them with two three-layer feedforward neural networks (one network to produce the rewards, and another one to produce the MARs). Both neural networks have the same structure: two input neurons, five hidden neurons, and a single output neuron. The first neural network is used when playing the role of partner: it senses an individual’s own productivity and that of her decision maker, and produces the reward as output. The second network is used when playing the role of decision maker: it senses an individual’s own productivity and that of her partner, and produces the MAR as output.

Each neuron in the networks computes an output signal of value

$$\begin{array}{c} output=\frac{1}{1+e^{(}-input)} \end{array}$$ (4)

with *input* being a linear combination of the outputs of the neurons of the previous layer and the related synaptic weights. This is a function routinely used in evolutionary robotics [43], but see [44, 45] for a discussion of the impact of other connectivities. Synaptic weights can take values from the interval [-5, 5], and are randomly drawn from a uniform law covering this interval at the start of the simulation.

Each network has its own set of synaptic weights, that are transmitted genetically. Because evolution now operates on these weights, and not on rewards or MARs directly, individuals can now evolve a reaction norm. They can evolve a function that produces outputs even from inputs they have never encountered before (i.e., individuals of new productivities). This property of neural networks is important in our case, because equity is precisely a relationship between two quantities, contribution and reward. Seeing whether natural selection will be able to recreate the same relationship of proportionality between contribution and reward using simple neural networks is thus of great interest. All other methodological details for Simulations Set 2 are the same as in Simulations Set 1.

### Simulations set 3

As a final test of the robustness of our model, we test whether natural selection also favors divisions proportional to contributions when contribution is measured in terms of time invested into cooperation (instead of productivity). We present the details of these simulations and its results in SI section 1.1.

## Results

We first present the results for Simulations Set 1. Parameter values used to obtain the figures are summarized in Table 1. Reasonable deviations from these values do not alter the results. Moreover, analytical results confirm the results of Simulation Set 1 (see SI section 2).

**Fig 1:**
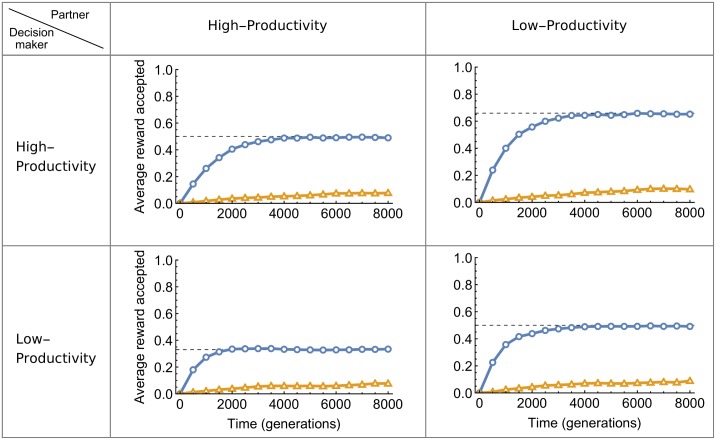
Evolution of the average rewards accepted in cooperative interactions according to the productivity of the decision maker and the partner. High-productivity individuals produce twice as much resources as low-productivity individuals. When partner choice is not costly, rewards evolve to match the decision maker’s relative contribution. Dashed lines represent the expected reward in the analytical model. The evolution of MARs is visually undistinguishable from the evolution of rewards and thus not represented.

We present the case where high-productivity individuals are able to produce twice as much resources as low-productivity individuals (*a* = 1, *b* = 2). Fig 1 shows the evolution of rewards *r* accepted by decision makers across generations. Rewards increase in all possible combinations of productivities, when partner choice is not costly (circle markers). If we focus on rewards accepted by high-productivity decision makers with low-productivity partners (Fig 1, upper-right panel), simulations show that at the evolutionary equilibrium, low-productivity partners have to give exactly 66% of the total resource produced to their high-productivity decision makers. This reward is exactly proportional to the relative contribution of each individual, as high-productivity individuals produce 66% of the total shared resource when *a* = 1 and *b* = 2. Similarly, high-productivity partners give only 33% to low-productivity decision makers, a reward which low-productivity decision makers accept, as it corresponds to their relative contribution (Fig 1, lower-left panel, circle markers). Finally, both high-productivity and low-productivity individuals give each other exactly 50% of the total resource when they meet as a pair, reflecting the fact that proportionality means equal division when contributions are equal (Fig 1, upper-left and lower-right panels). This pattern of divisions is confirmed by the analytical model (dashed lines in Fig 1, and see SI section 2), and divisions proportional to contribution also evolve when contribution is measured in terms of time invested into cooperation instead of productivity (see SI section 1.1).

**Fig 2:**
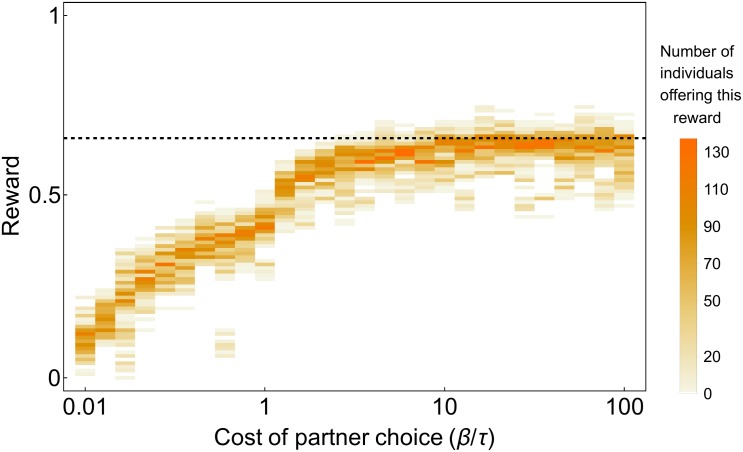
Distribution of rewards offered by low-productivity individuals to high-productivity individuals in the last generation of an 8,000-generation simulation, for different levels of partner choice cost (higher values of $\frac{\beta }{\tau }$ represent lower costs). High-productivity individuals’ relative contribution compared to low-productivity individuals is 0.66, so the dashed line represents the expected equitable distribution. This distribution can only be reached when partner choice is not costly ($\frac{\beta }{\tau }$ is high).

By comparing simulations with a low and a high $\frac{\beta }{\tau }$ ratio, Fig 1 also emphasizes the critical importance of partner choice for proportional rewards to evolve. When we decrease the $\frac{\beta }{\tau }$ ratio, individuals spend more time looking for new partners and thus the cost of changing partners is increased. In this situation, rewards remain very low over generations and never rise towards proportionality, regardless of differences in productivity (Fig 1, triangle markers). For instance, even if low-productivity partners produce less than half of the resources when they cooperate with high-productivity decision makers, they keep most of the resources for themselves when partner choice is costly. Fig 2 shows the distribution of rewards given by low-productivity individuals to high-productivity individuals at the end of an 8,000-generation simulation, for different values of the $\frac{\beta }{\tau }$ ratio. Proportional rewards of 66% can only evolve when $\frac{\beta }{\tau }$ is large, showing again that without partner choice, proportionality cannot evolve.

**Fig 3:**
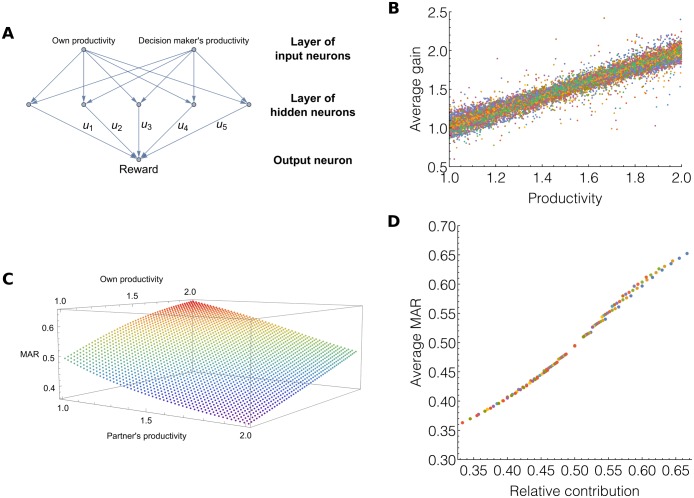
Evolution of equitable rewards made by neural networks working on a continuum of productivities. **A:** Schematic representation of the neural networks that make rewards. Networks take each individual’s productivity as inputs and produce the reward as output. The u’s represent synaptic weights on which evolution takes place. **B:** 15,000 individuals and their lifelong average gain plotted against their productivity. **C:** Average MARs produced by the neural networks of 15,000 individuals after 8,000 generations, for different values of the input neurons. The more an individual produces and the less the partner produces, the larger the individual’s MAR. **D:** Average MARs produced by 15,000 neural networks plotted against the relative contribution of the bearer of the network.

The results of Simulation Set 2 confirm this pattern. With a continuum of productivities in the population (between 1 and 2), rewards still respect proportionality at the evolutionary equilibrium. Each individual who enters an interaction is rewarded with an amount of resources exactly equal to her productivity (Fig 3B). As explained in the methods section, neural networks have two inputs: an individual’s own contribution and her partner’s (or decision maker’s) contribution. It is thus possible to represent the output of a network on a 3D plot, shown in Fig 3C. To plot this figure, we extracted the synaptic weights of the neural networks producing MARs for 15,000 individuals, at the last generation of 30 different simulation runs. We averaged the value of the networks’ outputs over those 15,000 individuals. Fig 3C shows that the networks evolved to produce MARs that are proportional to their bearer’s relative contribution (Fig 3C and 3D, and see SI section 3.2). The higher the decision maker’s productivity, and the lower the partner’s productivity, the more demanding the decision maker becomes.

## Discussion

We modelled a population of individuals choosing each other for cooperation. When different contributions to cooperation are made, resource divisions proportional to contributions evolve. Individuals producing more resources or investing more time into cooperation receive more resources than individuals producing or investing less. Asking for divisions that match one’s own contribution, and proposing such divisions to others, constitutes the best strategy when partner choice is possible. In other terms, a preference for equity maximizes fitness in an environment where individuals can choose their cooperative partners.

It is important to note that our results cannot be summarized as “a preference for equity helps individuals to be chosen as a partner” or “a preference for equity helps avoid interactions with selfish partners.” This is only half of the story. If the point were only to be chosen as a partner, the best strategy would be to be as generous as possible, an outcome which is sometimes observed in models inspired by competitive altruism theories [46]. The point here is rather to be chosen as a partner while at the same time avoiding exploitation by being over-generous. Our model clearly shows that the best strategy to solve this problem is to give proportionally to the other’s contribution—not less, but also not more. Equity is the result of a trade-off between two evolutionary pressures which work in opposite directions: the pressure to keep being chosen, but also the pressure to choose wisely.

This last point is better understood by looking at the precise mechanism through which proportionality evolves. The key factor determining divisions of resources at the evolutionary equilibrium are the opportunity costs of each individual. Opportunity costs represent the benefits an individual renounces to when she makes a choice. From an evolutionary point of view, it is trivial that an individual will want to make the best choices possible to minimize her opportunity costs. Hence, the best strategy to keep being chosen as a cooperative partner is to compensate others’ opportunity costs: when individual A agrees to interact with individual B, individual B should give A something equal to A’s opportunity costs at the time of making the decision (and vice versa). This is exactly why high-productivity individuals get more in our model: high-productivity individuals have larger opportunity costs than low-productivity individuals. Suppose that low-productivity individuals produce 1 unit of a resource whereas high-productivity individuals produce 2. High-productivity individuals thus have the possibility to produce 4 resources when they interact with other high-productivity individuals, leaving them with 2 resources on average (see exactly why in SI section 3.1). 2 resources is thus the opportunity cost of high-productivity individuals when they agree to cooperate with low-productivity individuals. Thus, if low-productivity individuals want to be good partners, they will have to compensate high-productivity individuals’ opportunity costs and give them exactly 2 resources (out of 3 produced), which will result in a proportional offer of 66%. But low-productivity individuals should not give more neither, because they also have access to interactions in which they could gain 1 unit on average (when they cooperate with other low-productivity individuals). In other words, low-productivity individuals have opportunity costs of 1, and should thus not accept divisions leaving them with less than 1. Our current model and previous papers on the subject [26, 27, 40] push forward the idea that the sense of fairness is a psychological mechanism evolved to compensate others’ opportunity costs and minimize one’s own opportunity costs. This characterization only comes from models investigating fairness in distributive situations though, so it would be interesting to see if it holds in more diverse, non-distributive situations.

Our model has several limitations, which need to be acknowledged. First, while we suppose that individuals choose each other based on their reputation, we do not explicitly model the formation of this reputation. Individuals automatically know the reputation of others and this reputation is reliable. It could be interesting to relax this assumption, especially because reputation formation (through communication for instance) might be an important point that distinguishes humans from non-human primates. Second, the population we model does not match the hunter-gatherer population in the sense that it is not structured. This is important because a structure, such as camps or family units, could potentially affect opportunities to choose partners. Finally, it might be interesting to model the evolution of fairness in a wider range of cooperative interactions than we have considered here (outside distributive situations for instance). All of these assumptions should be relaxed in future studies.

Partner choice is not the only evolutionary mechanism postulated to lead to the evolution of fairness in the literature. Some authors have argued that fairness could be explained by empathy [24], spite [25, 47, 48], “noisy” processes such as drift or learning mistakes [23, 49], the existence of a spatial population structure [50, 51], or alternating offers [52, 53]. But as we explained in the introduction, all of these models equate fairness with equality, and it is thus unknown whether they can explain a more general case. Testing whether those models pass the “equity test” will be an excellent way to compare and decide between these models, a necessary undertaking that has been largely neglected. The extensive literature on “bargaining” in economics (Binmore, 1986; Binmore, 1998; Alexander, 2000) was also more focused on the case in which players are in a symmetric position, and usually did not investigate proportional bargaining solutions. An exception is the work by Kalai (1977) (although Binmore, 2005 also mentions the problem p. 31), who shows that individuals will compromise in different bargaining situations so as to keep their proportions of utility gains fixed. But, as Kalai recognizes it himself (P11), “a more difficult problem is to find what these proportions should be”. This is precisely where we make a contribution: we show that when individuals evolve in biological markets, these proportions are automatically determined by the other encounters individuals can make. In other words, one could rephrase our model as showing that individuals can bargain based on their outside options (or opportunity costs), but contrarily to what has been done before, we do not fix exogenously those outside options. Rather, outside options emerge endogenously from all the encounters individuals can make in the population.

Talking about bargaining theory suggests alternative interpretations of our model. It might be argued that human fairness is the result of bargaining at the proximal level, the result of rational cognitive processes. We argue instead that the “bargaining” already took place at the ultimate level by means of natural selection, and that the result of this bargaining is the existence of a genuine sense of fairness which “automatically” makes humans prefer equitable strategies. This hypothesis does not exclude the possibility that humans are also capable of consciously bargaining based on their opportunity costs, but this behavior would not be the product of an evolved sense of fairness. While our model bears a great resemblance to historical market models [54] and other models in economics in which fair outcomes have sometimes been observed [52, 55], we emphasize that the markets we model are ultimate biological markets [56, 57]. This is not just an empty terminological variation: locating markets at the ultimate level has important implications for our understanding of the psychological mechanisms underlying fairness. Among other things, it allows us to understand why fairness does not seem to be based on self-interest at the psychological level even if fairness evolved for self-interested reasons [9, 58].

Another alternative interpretation of our model remains. One could agree that fairness judgments are based on simple automatic rules rather than complex conscious calculations, but argue that those rules could have evolved culturally rather than biologically. This is not an issue that can be settled theoretically, as the same models can always be interpreted as instances of biological or cultural evolution. To date, we definitely lack empirical data to answer this question with certainty, but the idea of a biologically evolved sense of fairness is not made absurd by the existing data. As early as the age of 12 months, children react to inequity [59–61], equity has been identified in many cultures around the word [12, 13], and children reject conventional rules when they violate principles of fairness [62]. We do not take experiments on inequity aversion in non-human primates as evidence for a biologically evolved sense of fairness, as the negative reactions to inequity observed so far can still be interpreted in more parsimonious ways (see [63] for a review and [64] for methodological issues). Nonetheless, those experiments remind us that many researchers expect that prosocial behaviors traditionally associated with the existence of human institutions, religions, or cultural artefacts can also evolve biologically. In fact, Robert Trivers himself recognized that the most important implication of his seminal paper on the evolution of reciprocity [58] was that “it laid the foundation for understanding how a sense of justice evolved” [65].

The existence of intercultural and interindividual variations in fairness judgements [10, 16, 66] is sometimes taken as evidence against their biological origin. This criticism is generally ill-founded, as evolutionary explanations have no particular difficulty accommodating variation [67]. In the case of fairness, it is important to remember that what our model predicts is not the evolution of a fixed judgement but the evolution of an algorithm, an information-processing mechanism [67]. This is particularly evident in our extended simulations where the evolving unit is a neural network, precisely a special type of algorithm. This algorithm works on inputs (contributions) to produce outputs (divisions of resources), and here lies an important source of variability, because inputs can vary across cultures and individuals while the algorithm remains the same. For instance, measurements of contributions are affected by beliefs (“How long do I think it takes to harvest this quantity of food?”). If contribution was the only input in our model, in real-life more parameters can affect the algorithm’s inputs, such as general knowledge (“Is this person not productive because she is sick?”) or individual interpretations of the situation (“Are we engaged in a communal interaction? A joint venture? A market exchange?”). This last point could explain why even in carefully controlled environments, where there is little ambiguity about the source of inequalities, there is still heterogeneity in fair behaviors, with some people behaving as egalitarians, others as meritocrats, and others still as libertarians [10, 68].

In fact, while interindividual and intercultural variations have crystallized the debate, intra-individual variation can also be observed even in Western countries. In some situations we behave as meritocrats, requiring pay for each additional hour of presence at work [2, 8], whereas the next day on a camping trip with strangers we behave more like egalitarians, without constant monitoring and bookkeeping of our contributions and those of others [69]. Neither our brain (the algorithm) nor our culture has changed in the meantime. What has changed is the way we interpret the situation (part of the input to the algorithm). This idea needs to be developed more formally, and we do not suggest that it is the only way to explain variation, but it may constitute a fruitful avenue of research.

Another interesting question is the prevalence of equity in traditional societies. We have mentioned anthropological records of distributions according to effort [13, 70], but it is also well known that hunter-gatherers transfer meat in a way that not does not seem to respect equity. This type of interaction has been called “generalized reciprocity” by [71] and also seems to match [72]’s notion of a “communal sharing” system. There are at least two mutually compatible ways to reconcile this observation with the predictions of our model. The first is to recognize that equity can be limited by other factors, for instance diminishing returns to consumption [73]. People could stop caring about equity when they become satiated or when they receive little additional value from consuming one more unit of benefits. The second is to consider that even in generalized reciprocity good hunters are rewarded with more benefits, but those benefits are delayed. This hypothesis has received support recently from findings showing that generous hunters and hard workers are central in the social networks of small-scale societies [74, 75]. In this last perspective, our model should not be taken at face value as predicting the evolution of strict equity with immediate input/output matching, but more generally as input/output matching over a long time and across different cooperative activities (“generalized equity”).

We conclude by noting that proportionality is important in distributive justice but is also a cornerstone of institutional justice, wherein offenders are punished in proportion to the severity of their crimes [76, 77]. It is also central to the morality of many religions, in which rewards and punishments are made proportional to good and bad deeds by supernatural entities or forces [78]. Although this is only speculation at present, our results may thus also explain why historically recent cultural domains such as penal justice and moral religions insist on the principle of proportionality: retributive punishment and supernatural justice may reflect our evolved desire for proportionality.

## Supporting information

S1 File Simulation procedures, analytical model and supplementary discussion. (PDF)
