# Correction: On the evolutionary origins of equity

**DOI:** 10.1371/journal.pone.0184459

**Published:** 2017-09-01

**Authors:** Stéphane Debove, Nicolas Baumard, Jean-Baptiste André

Three references that were cited in fifth sentence of the fifth paragraph in the Discussion were omitted from the Reference section. The sentence is: The extensive literature on “bargaining” in economics (Binmore, 1986; Binmore, 1998; Alexander, 2000) was also more focused on the case in which players are in a symmetric position, and usually did not investigate proportional bargaining solutions.

The references are:

Binmore, K., Rubinstein, A., & Wolinsky, A. (1986). The Nash bargaining solution in economic modelling. *RAND Journal of Economics*, *17*(2), 176–188. http://doi.org/10.2307/2555382

Binmore, K. (1998). The evolution of fairness norms. *Rationality and Society*, *23*, 151–173. Retrieved from http://rss.sagepub.com/content/10/3/275.short

Alexander, J. M. (2000). Evolutionary Explanations of Distributive Justice. *Philosophy of Science*, *67*(3), 490–516. http://doi.org/10.1086/392792

A reference that was cited in the sixth sentence of the fifth paragraph in the Discussion was omitted from the References section. The sentence is: An exception is the work by Kalai (1977) (although Binmore, 2005 also mentions the problem p. 31), who shows that individuals will compromise in different bargaining situations so as to keep their proportions of utility gains fixed.

The reference is: Kalai, E. (1977). Proportional Solutions to Bargaining Situations: Interpersonal Utility Comparisons. *Econometrica*, *45*(7), 1623–1630. http://doi.org/10.2307/1913954
