# Supplementary material for: On the evolutionary origins of equity
Source: PLoS One. 2017 Mar 21;12(3):e0173636. doi: 10.1371/journal.pone.0173636 (PMC5360236; doi:10.1371/journal.pone.0173636)
Supplement: S1 File — (PDF) [file pone.0173636.s001.pdf]

Supplementary information for manuscript

On the evolutionary origins of equity

1 Simulation procedures

1.1 Simulations Set 3: contribution through time invested

1.1.1 Methods

Having a higher productivity is only one way to contribute more to a cooperative interaction. Another natural way is to spend more time to amass resources. To test the robustness of our partner choice mechanism, we thus created a third set of simulations in which there are no more differences of productivity between individuals, but one of the two individuals in a cooperating dyad has to invest m times more time than her partner. We thus model the possibility that there is a cooperative role more time-consuming than the other. In practice, we model this by randomly attributing a "high investment of time" role to the partner or the decision maker when an encounter takes place. The decision maker then decides whether or not she wants to cooperate with her partner based on her partner's reputation for a given level of investment into cooperation. Each individual is thus characterized by 4 genetic variables, two r_{kl} and two MAR_{kl}, with k and $l \in \{H, L\}$, denoting an individual's time investment (H = High, L = Low). If the partner is accepted, individuals share a constant resource of size 1 at each unit of time, and the end of the interaction is determined in the same way than in Simulations Set 1, through a constant split rate τ. When a split happens though, the individual who needs to invest more time is prevented to encounter new individuals for a length of time equal to $(m - 1) *$ (the length of the interaction). Because this individual is prevented to encounter other individuals during this period, one can interpret this period as a period in which this individual is still investing time into the previous interaction.

All other methodological details for Simulations Set 3 are the same as in Simulations Set 1. In particular, we start from a population of individuals giving zero reward even when they

Average reward accepted	By individuals investing half as much time	By individuals investing twice as much time
	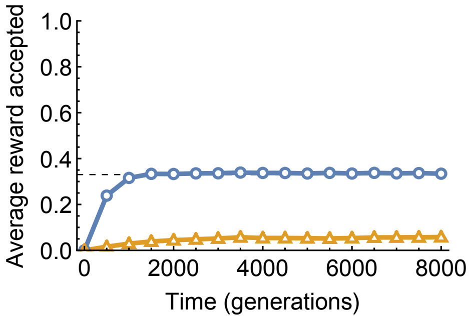	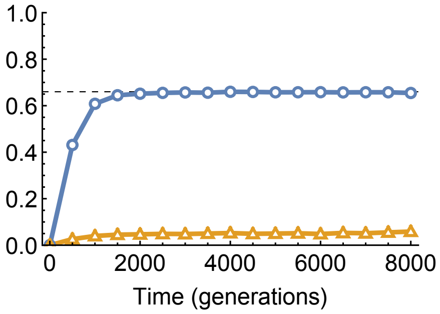

Figure SM1: Evolution of the average reward accepted, depending on whether partners invest twice as much or half as much time into cooperation. Individuals investing twice as much time receive twice as much resources at equilibrium, and vice-versa.

invest less time into cooperation, and observe what will be the relationship between contribution (time invested) and rewards at the evolutionary equilibrium.

1.1.2 Results

Simulations Set 3 show that proportional rewards also evolve when individuals differ not by their productivity but by the time they invest in cooperation (Fig SM1). Setting $m = 2$, one individual of the pair has to invest twice as much time as the other. When the decision maker invests twice as much time, the partner agrees to reward him with 66% of the total resource at the evolutionary equilibrium, when partner choice is not costly. Conversely, when decision makers invest half as much time as their partner, they accept rewards of 33% only, showing that the fitness-maximizing strategy in this situation is to accept rewards proportional to each partner's relative time investment.

2 Analytical model.

We developed an analytical model to model the situation where individuals differ by their productivity (but not effort), and where only two productivities coexist in the population. The analytical model incorporates all of the features of the simulations, but with one simplification: we assume that the total number of interactions accepted per unit of time is the same for each individual. With this assumption, rejecting an opportunity to cooperate does not compromise the chances of cooperating later, but on the contrary grants new opportunities. This situation is analogous to the condition where $\frac{\beta}{\tau}$ tends towards infinity in the simulations: social opportunities are plentiful at the scale of the length of interactions. When individuals reject an interaction, however, they are forced to postpone their social interaction to a later encounter. We assume that this entails an explicit cost expressed as a discounting factor δ ($0 \leq \delta < 1$). If we call the average payoff of an individual of productivity i G_i, then δG_i will be the average expected payoff in the next interaction after rejecting an offer. When δ equals 1, refusing an interaction carries no cost; when δ equals 0, refusing an interaction will result in zero payoff from the next interaction. In practice, we will neglect the case where δ equals 1, as it leads to artefactual results (see below).

The assumption that only partners can decide of the division in our model is necessary so that the evolution of fairness is not explained trivially. When only one individual can decide, natural selection favors selfishness [1]. This is easy to understand. On the one hand, whatever reward a partner suggests, accepting it brings a greater gain than rejecting it for the decision maker. Therefore, in all cases, natural selection favors indiscriminate partners, with decision makers taking whatever benefits are made available to them. On the other hand, and as a result, selection favors stingy partners, offering the minimal possible amount. Because decision makers are in such an inferior bargaining position, in the following analysis we will focus on

decision makers'—and not partners'—payoffs. A decision maker receiving a large share of the resource is a strong indication that there are evolutionary forces at work against the expected partners' selfishness.

All our analyses assume that (i) individuals enter the population at a constant rate, (ii) evolution is slow compared to an individual's lifespan (and thus) (iii) mutations are rare, and that (iv) there is no recombination between genetic traits (p_{ij} and q_{ij}). As a consequence of (i) and (ii), the composition of the population does not change during an individual's life. As a consequence of (iii) and (iv), at any evolutionary equilibrium, all the strategies present in the population must reach the same payoff for individuals of a given strength (only a high mutation rate or recombination rate could continuously re-introduce maladaptive strategies in the population, yielding a variance of payoffs at each generation).

Here we ask the same question answered in the main paper through simulations: how will the behavioural traits r_{ij} and MAR_{ij} (i and $j \in \{HP, LP\}$) evolve in an environment where LP and HP individuals coexist and share resources? As a reminder, MAR_{LPHP} reads as "the minimum reward that a LP individual will accept from a HP individual," and r_{HPLP} as "the reward a HP individual will give to a LP individual."

Following the precise evolutionary dynamics of the system to answer this question is quite a complex challenge, in particular due to epistasis phenomena. The low fitness benefits brought by a reward r can be compensated by high benefits from an acceptance threshold MAR, or small benefits obtained in interactions with individuals of one productivity could be compensated by high benefits received in interactions with the other productivity, generating linkage disequilibrium [2]. But as in [1], it is easier to derive simple conditions on the payoff an individual would or would not have an interest in accepting at the evolutionary equilibrium.

2.1 Solving the system

The reasoning is more normative than descriptive, as we consider a situation in which the equilibrium has already been reached, and derive constraints on the values of traits that individuals should display at the equilibrium. To derive the payoff a LP individual should receive from a HP individual at the evolutionary equilibrium, we need to consider four arguments:

1. **All individuals with the same productivity must gain the same payoff.** At the equilibrium, all HP individuals should gain the same payoff G_{HP} per interaction (otherwise it wouldn't be an equilibrium), and the same is true for LP individuals. We thus only have two average payoffs in the population at the equilibrium. The average payoff of a HP individual is labeled G_{HP}, and that of a LP individual is written G_{LP}.

2. **Every individual of productivity i accepts exactly δG_i**, with $i \in \{HP, LP\}$. If an individual's average payoff is G_i, his expected payoff in the next interaction (if the current interaction is refused) will be δG_i. As a consequence, a decision maker should never refuse a reward that is above the corresponding δG_i, but should always refuse rewards that are below this level. At the equilibrium, because rewards from partners should evolve toward the minimum that decision makers will accept, individuals will always demand and accept exactly δG_i, no matter who they are interacting with (regardless of their partner's productivity). We thus have:

$$\begin{cases} \text{MAR}_{HPHP} = \delta G_{HP} \\ \text{MAR}_{HPLP} = \delta G_{HP} \\ \text{MAR}_{LPLP} = \delta G_{LP} \\ \text{MAR}_{LPHP} = \delta G_{LP} \end{cases} \tag{1}$$

3. **Partners give their decision makers what they want at the evolutionary equilibrium, as long as $\frac{a}{b} > \frac{\delta(x-1)}{\delta x - 2}$.**

Knowing (1) and (2), it can be shown that partners are always better off giving their decision makers what they "ask for" (δG_i) at the evolutionary equilibrium, as long as $\delta < 1$. The reasoning is as follows.

Suppose that at the evolutionary equilibrium, all LP individuals refuse to give HP individuals what they ask for, namely δG_{HP} (but all other demands are satisfied). The average social payoff of a LP individual in this population is then

$$G_{\mathrm{LP}} = (1-x)\left(\frac{\delta G_{\mathrm{LP}}}{2} + \frac{\delta G_{\mathrm{LP}}}{2}\right) + \frac{1}{2}x\,(a+a) \tag{2}$$

with x the proportion of LP individuals in the population and a the productivity of LP individuals. G_{LP} can be decomposed into three terms: an average payoff obtained in interactions with other LP individuals $\frac{1}{2}(a+a)$, an average payoff obtained in interactions with HP individuals when HP individuals play the role of decision makers (in this case, under our hypothesis the reward will be rejected and the LP individual's payoff will be discounted by δ), and, finally, an average payoff obtained in interactions with HP individuals when HP individuals are partners (the LP individual's MAR is met, so they gain δG_{LP}).

Similarly, the payoff of a HP individual in this population is

$$G_{\mathrm{HP}} = x\left(\frac{\delta G_{\mathrm{HP}}}{2} + \frac{1}{2}\left(-\delta G_{\mathrm{LP}} + b + a\right)\right) + \frac{1}{2}(1-x)\,(b+b) \tag{3}$$

with b the productivity of HP individuals. Solving the system composed of equations (2) and (3) gives us an expression for G_{HP} and G_{LP}. The question we need to answer now is the following: what would happen if, in such a population, a mutant LP individual decided to accept to give HP individuals what they want? Upon meeting a HP individual

and being assigned the role of partner, this mutant would gain $a+b-\delta G_{HP}$ (the resource to be shared minus the demand of a HP individual) instead of just δG_{LP} (the average payoff being discounted). Knowing G_{LP} and G_{HP}, it is easy to show that it is never possible that $\delta G_{LP} \geq a+b-\delta G_{HP}$ as long as $\delta < 1$. In other words, at the evolutionary equilibrium, it is impossible that all LP individuals refuse to offer δG_{HP} to HP individuals, because they would gain more from doing so.

What if there was some polymorphism in the population such that only *some* LP individuals refuse to give HP individuals what they ask for? The average social payoff of those LP individuals is still written the same as in equation (2). But because we know that at the evolutionary equilibrium all individuals with the same productivity must gain the same payoff, the payoff of all LP individuals will be the same, regardless of phenotype. The coexistence of two types of LP individuals in the population would imply that $\delta G_{LP} = a + a - \delta G_{HP}$ (the payoff of the two types of LP individuals in the position of partner when paired with HP individuals is equal), but as we showed above, this is not possible as long as $\delta < 1$. As a consequence, it is not only impossible that *all* LP individuals refuse to give HP individuals what they want at the evolutionary equilibrium, it is also impossible that *some* LP individuals refuse to give HP individuals what they want as long as $\delta < 1$.

Following the same reasoning, it can be shown that it is not possible for some individuals (of any productivity) to refuse to give their social partner (of any productivity) what they ask for at the evolutionary equilibrium as long as $\frac{a}{b} > \frac{\delta(x-1)}{\delta x-2}$ (see SM section 2.2). When $\frac{a}{b} \leq \frac{\delta(x-1)}{\delta x-2}$, it is possible that LP individuals refuse to give other LP individuals what they ask for. This condition reflects the fact that if the difference of productivity between HP and LP individuals is too large, it is more beneficial for LP individuals to

interact with HP individuals than with LP individuals. As we will see though, this is only possible when partner choice is costly. Moreover, as long as $\frac{a}{b} > 0.5$, as is the case in our simulations, it is not worth it for LP individuals to refuse to interact with other LP individuals, and so all partners will give their decision makers what they want at the evolutionary equilibrium.

If $\frac{a}{b} > \frac{\delta(x-1)}{\delta x-2}$, we can thus write:

$$\begin{cases} r_{HPHP} = \delta G_{HP} \\ r_{HPLP} = \delta G_{LP} \\ r_{LPLP} = \delta G_{LP} \\ r_{LPHP} = \delta G_{HP} \end{cases} \tag{4}$$

and if $\frac{a}{b} \leq \frac{\delta(x-1)}{\delta x-2}$, we can thus write:

$$\begin{cases} r_{HPHP} = \delta G_{HP} \\ r_{HPLP} = \delta G_{LP} \\ r_{LPHP} = \delta G_{HP} \end{cases} \tag{5}$$

4. **$\frac{a}{b} > \frac{\delta(x-1)}{\delta x-2}$, no offer is never refused**

If $\frac{a}{b} > \frac{\delta(x-1)}{\delta x-2}$, from step 3. it directly results that no reward is ever rejected at the evolutionary equilibrium, because each partner's reward is exactly equal to the decision maker's MAR, and thus each reward is accepted. If no reward is ever refused, the average payoff of LP and HP individuals respectively can be written as:

$$\begin{cases} G_{\mathrm{LP}} = (1-x)\left(\frac{1}{2}\left(-\delta G_{\mathrm{HP}} + b + a\right) + \frac{\delta G_{\mathrm{LP}}}{2}\right) + \frac{1}{2}x\left(a+a\right) \\ \\ G_{\mathrm{HP}} = x\left(\frac{\delta G_{\mathrm{HP}}}{2} + \frac{1}{2}\left(-\delta G_{\mathrm{LP}} + b + a\right)\right) + \frac{1}{2}(1-x)\left(b+b\right) \end{cases} \tag{6}$$

Solving this system gives us an expression for G_{HP} and G_{LP} as a function of x and δ at the evolutionary equilibrium:

$$\begin{cases} G_{LP} = \frac{b(\delta-\delta x+x-1)+a((\delta-1)x-1)}{\delta-2} \\ \\ G_{HP} = \frac{b(\delta-\delta x+x-2)+(\delta-1)xa}{\delta-2} \end{cases} \tag{7}$$

From (5) and (8), it is straightforward to show that when δ tends toward 1 (partner choice is not costly), r_{LPHP} tends toward b. That is, when partner choice is not costly, even if LP individuals are in the strategically dominant position of partner, at the evolutionary equilibrium they offer HP individuals an amount that is exactly equal to their productivity b. In percentage, this corresponds to an offer proportional to the relative contribution of each individual: LP individuals offer HP individuals $\frac{b}{b+a} * 100$ % of the total resource to be shared.

Similarly, it can be shown that when δ tends toward 1, LP individuals offer other LP individuals a resources, HP individuals offer other HP individuals b resources, and HP individuals offer LP individuals a resources. At the equilibrium, when partner choice is not costly each individual is rewarded with an amount exactly equal to his contribution.

5. $\frac{a}{b} \leq \frac{\delta(x-1)}{\delta x-2}$**, all LP individuals refuse to interact with other LP individuals**

In this case, the average payoff of LP and HP individuals respectively can be written as:

$$\begin{cases} G_{\mathrm{LP}} = (1-x)\left(\frac{1}{2}\left(-\delta G_{\mathrm{HP}}+b+a\right)+\frac{\delta G_{\mathrm{LP}}}{2}\right)+\delta x G_{\mathrm{LP}} \\ \\ G_{\mathrm{HP}} = x\left(\frac{\delta G_{\mathrm{HP}}}{2}+\frac{1}{2}\left(-\delta G_{\mathrm{LP}}+b+a\right)\right)+\frac{1}{2}(1-x)\left(b+b\right) \end{cases} \tag{8}$$

Solving this system gives us an expression for G_{HP} and G_{LP} as a function of x and δ at the evolutionary equilibrium:

$$\begin{cases} G_{LP} = \frac{(x-1)((\delta-1)b+a(\delta x-1))}{\delta(x(\delta x-2)-1)+2} \\ \\ G_{HP} = \frac{b(\delta((x-1)x-1)-x+2)-(\delta-1)xa}{\delta(x(\delta x-2)-1)+2} \end{cases} \tag{9}$$

From (6) and (10), it is straightforward to show that when δ tends toward 1, the previous results hold: LP individuals offer HP individuals b resources, HP individuals offer other HP individuals b resources, and HP individuals offer LP individuals a resources.

2.2 Verification that partners are always better off giving their decision maker what they want at the evolutionary equilibrium, except when $\frac{a}{b} \leq \frac{\delta(x-1)}{\delta x-2}$

There are four hypothetical primary situations that need to be taken into account:

- A: when HP individuals are partners, they refuse to give other HP individuals what they want
- B: when HP individuals are partners, they refuse to give other LP individuals what they want
- C: when LP individuals are partners, they refuse to give other LP individuals what they want
- D: when LP individuals are partners, they refuse to give HP individuals what they want

These situations are not mutually exclusive, however, so the total number of possible situations is:

$\sum_{k=1}^{4} \binom{4}{k} = 15$

Situation D was already proven to be impossible at the evolutionary equilibrium in the previous section. We now show that the same holds for the 14 remaining situations, except in situation C. We give the expected social payoff of HP and LP individuals in each situation. We also give the condition that must be satisfied for each situation to be possible at the evolutionary equilibrium; it is then straightforward to show that, given our parameter values ($0 \leq x \leq 1$, $0 \leq \delta < 1$), this condition can never be satisfied.

Situation A:

- $G_{\text{LP}} = (1-x)\left(\frac{1}{2}\left(-\delta G_{\text{HP}} + b + a\right) + \frac{\delta G_{\text{LP}}}{2}\right) + \frac{1}{2}x\left(a + a\right)$
- $G_{\text{HP}} = x\left(\frac{\delta G_{\text{HP}}}{2} + \frac{1}{2}\left(-\delta G_{\text{LP}} + b + a\right)\right) + \delta(1-x)G_{\text{HP}}$
- Condition $-\delta G_{\text{HP}} + b + b \leq \delta G_{\text{HP}}$ impossible

Situation C:

- $G_{\text{LP}} = (1-x)\left(\frac{1}{2}\left(-\delta G_{\text{HP}} + b + a\right) + \frac{\delta G_{\text{LP}}}{2}\right) + \delta x G_{\text{LP}}$
- $G_{\text{HP}} = x\left(\frac{\delta G_{\text{HP}}}{2} + \frac{1}{2}\left(-\delta G_{\text{LP}} + b + a\right)\right) + \frac{1}{2}(1-x)\left(b + b\right)$
- Condition $-\delta G_{\text{LP}} + a + a \leq \delta G_{\text{LP}}$ impossible when $a > \frac{\delta(x-1)b}{\delta x - 2}$

Situation B:

- $G_{\text{LP}} = (1-x)\left(\frac{1}{2}\left(-\delta G_{\text{HP}} + b + a\right) + \frac{\delta G_{\text{LP}}}{2}\right) + \frac{1}{2}x\left(a + a\right)$
- $G_{\text{HP}} = x\left(\frac{\delta G_{\text{HP}}}{2} + \frac{\delta G_{\text{HP}}}{2}\right) + \frac{1}{2}(1-x)\left(b + b\right)$
- Condition $-\delta G_{\text{LP}} + b + a \leq \delta G_{\text{HP}}$ impossible

Situation A & C:

- $G_{\text{LP}} = (1-x)\left(\frac{1}{2}\left(-\delta G_{\text{HP}} + b + a\right) + \frac{\delta G_{\text{LP}}}{2}\right) + \delta x G_{\text{LP}}$

- $G_{\mathrm{HP}} = x\left(\frac{\delta G_{\mathrm{HP}}}{2} + \frac{1}{2}\left(-\delta G_{\mathrm{LP}} + b + a\right)\right) + \delta(1-x)G_{\mathrm{HP}}$
- Condition $-\delta G_{\mathrm{LP}} + a + a \leq \delta G_{\mathrm{LP}} \wedge -\delta G_{\mathrm{HP}} + b + b \leq \delta G_{\mathrm{HP}}$ impossible

Situation B & C:

- $G_{\mathrm{LP}} = (1-x)\left(\frac{1}{2}\left(-\delta G_{\mathrm{HP}} + b + a\right) + \frac{\delta G_{\mathrm{LP}}}{2}\right) + \delta x G_{\mathrm{LP}}$
- $G_{\mathrm{HP}} = x\left(\frac{\delta G_{\mathrm{HP}}}{2} + \frac{\delta G_{\mathrm{HP}}}{2}\right) + \frac{1}{2}(1-x)\left(b + b\right)$
- Condition $-\delta G_{\mathrm{LP}} + a + a \leq \delta G_{\mathrm{LP}} \wedge -\delta G_{\mathrm{LP}} + b + a \leq \delta G_{\mathrm{HP}}$ impossible

Situation C & D:

- $G_{\mathrm{LP}} = \delta x G_{\mathrm{LP}} + (1-x)\left(\frac{\delta G_{\mathrm{LP}}}{2} + \frac{\delta G_{\mathrm{LP}}}{2}\right)$
- $G_{\mathrm{HP}} = x\left(\frac{\delta G_{\mathrm{HP}}}{2} + \frac{1}{2}\left(-\delta G_{\mathrm{LP}} + b + a\right)\right) + \frac{1}{2}(1-x)\left(b + b\right)$
- Condition $-\delta G_{\mathrm{LP}} + a + a \leq \delta G_{\mathrm{LP}} \wedge -\delta G_{\mathrm{HP}} + b + a \leq \delta G_{\mathrm{LP}}$ impossible

Situation B & D:

- $G_{\mathrm{LP}} = (1-x)\left(\frac{\delta G_{\mathrm{LP}}}{2} + \frac{\delta G_{\mathrm{LP}}}{2}\right) + \frac{1}{2}x\left(a + a\right)$
- $G_{\mathrm{HP}} = x\left(\frac{\delta G_{\mathrm{HP}}}{2} + \frac{\delta G_{\mathrm{HP}}}{2}\right) + \frac{1}{2}(1-x)\left(b + b\right)$
- Condition $-\delta G_{\mathrm{HP}} + b + a \leq \delta G_{\mathrm{LP}} \wedge -\delta G_{\mathrm{LP}} + b + a \leq \delta G_{\mathrm{HP}}$ impossible

Situation A & D:

- $G_{\mathrm{LP}} = (1-x)\left(\frac{\delta G_{\mathrm{LP}}}{2} + \frac{\delta G_{\mathrm{LP}}}{2}\right) + \frac{1}{2}x\left(a + a\right)$
- $G_{\mathrm{HP}} = x\left(\frac{\delta G_{\mathrm{HP}}}{2} + \frac{1}{2}\left(-\delta G_{\mathrm{LP}} + b + a\right)\right) + \delta(1-x)G_{\mathrm{HP}}$
- Condition $-\delta G_{\mathrm{HP}} + b + a \leq \delta G_{\mathrm{LP}} \wedge -\delta G_{\mathrm{HP}} + b + b \leq \delta G_{\mathrm{HP}}$ impossible

Situation A & B:

- $G_{\mathrm{LP}} = (1-x)\left(\frac{1}{2}\left(-\delta G_{\mathrm{HP}} + b + a\right) + \frac{\delta G_{\mathrm{LP}}}{2}\right) + \frac{1}{2}x\left(a + a\right)$
- $G_{\mathrm{HP}} = \delta(1-x)G_{\mathrm{HP}} + x\left(\frac{\delta G_{\mathrm{HP}}}{2} + \frac{\delta G_{\mathrm{HP}}}{2}\right)$
- Condition $-\delta G_{\mathrm{HP}} + b + b \leq \delta G_{\mathrm{HP}} \wedge -\delta G_{\mathrm{LP}} + b + a \leq \delta G_{\mathrm{HP}}$ impossible

Situation A & C & D:

- $G_{\mathrm{LP}} = \delta x G_{\mathrm{LP}} + (1-x)\left(\frac{\delta G_{\mathrm{LP}}}{2} + \frac{\delta G_{\mathrm{LP}}}{2}\right)$
- $G_{\mathrm{HP}} = x\left(\frac{\delta G_{\mathrm{HP}}}{2} + \frac{1}{2}\left(-\delta G_{\mathrm{LP}} + b + a\right)\right) + \delta(1-x)G_{\mathrm{HP}}$
- Condition $-\delta G_{\mathrm{LP}} + a + a \leq \delta G_{\mathrm{LP}} \wedge -\delta G_{\mathrm{HP}} + b + b \leq \delta G_{\mathrm{HP}} \wedge -\delta G_{\mathrm{HP}} + b + a \leq \delta G_{\mathrm{LP}}$ impossible

Situation A & B & C:

- $G_{\mathrm{LP}} = (1-x)\left(\frac{1}{2}\left(-\delta G_{\mathrm{HP}} + b + a\right) + \frac{\delta G_{\mathrm{LP}}}{2}\right) + \delta x G_{\mathrm{LP}}$
- $G_{\mathrm{HP}} = \delta(1-x)G_{\mathrm{HP}} + x\left(\frac{\delta G_{\mathrm{HP}}}{2} + \frac{\delta G_{\mathrm{HP}}}{2}\right)$
- Condition $-\delta G_{\mathrm{LP}} + a + a \leq \delta G_{\mathrm{LP}} \wedge -\delta G_{\mathrm{HP}} + b + b \leq \delta G_{\mathrm{HP}} \wedge -\delta G_{\mathrm{LP}} + b + a \leq \delta G_{\mathrm{HP}}$ impossible

Situation B & C & D:

- $G_{\mathrm{LP}} = \delta x G_{\mathrm{LP}} + (1-x)\left(\frac{\delta G_{\mathrm{LP}}}{2} + \frac{\delta G_{\mathrm{LP}}}{2}\right)$
- $G_{\mathrm{HP}} = x\left(\frac{\delta G_{\mathrm{HP}}}{2} + \frac{\delta G_{\mathrm{HP}}}{2}\right) + \frac{1}{2}(1-x)\left(b + b\right)$
- Condition $-\delta G_{\mathrm{LP}} + a + a \leq \delta G_{\mathrm{LP}} \wedge -\delta G_{\mathrm{HP}} + b + a \leq \delta G_{\mathrm{LP}} \wedge -\delta G_{\mathrm{LP}} + b + a \leq \delta G_{\mathrm{HP}}$ impossible

Situation A & B & D:

- $G_{\mathrm{LP}} = (1-x)\left(\frac{\delta G_{\mathrm{LP}}}{2} + \frac{\delta G_{\mathrm{LP}}}{2}\right) + \frac{1}{2}x\left(a + a\right)$

- $G_{\mathrm{HP}} = \delta(1-x)G_{\mathrm{HP}} + x\left(\frac{\delta G_{\mathrm{HP}}}{2} + \frac{\delta G_{\mathrm{HP}}}{2}\right)$
- Condition $-\delta G_{\mathrm{HP}} + b + b \leq \delta G_{\mathrm{HP}} \wedge -\delta G_{\mathrm{LP}} + b + a \leq \delta G_{\mathrm{HP}} \wedge -\delta G_{\mathrm{HP}} + b + a \leq \delta G_{\mathrm{LP}}$ impossible

Situation A & B & C & D:

- $G_{\mathrm{LP}} = \delta(1-x)G_{\mathrm{LP}} + \delta x G_{\mathrm{LP}}$
- $G_{\mathrm{HP}} = \delta(1-x)G_{\mathrm{HP}} + \delta x G_{\mathrm{HP}}$
- Condition $-\delta G_{\mathrm{HP}} + b + b \leq \delta G_{\mathrm{HP}} \wedge -\delta G_{\mathrm{LP}} + b + a \leq \delta G_{\mathrm{HP}} \wedge -\delta G_{\mathrm{LP}} + a + a \leq \delta G_{\mathrm{LP}} \wedge -\delta G_{\mathrm{HP}} + b + a \leq \delta G_{\mathrm{LP}}$ impossible

As explained in the previous section, the verification that it is not possible for *some* (but not all) individuals not to interact with other individuals at the evolutionary equilibrium (in case of polymorphism) is already implied by the use of not strict inequalities.

3 Supplementary discussion

3.1 Opportunity costs

In the main article, we explain that when high-productivity individuals are assessing a low-productivity individual's reward, they have opportunity costs (or "outside options") of 2 because they expect to receive 2 with other high-productivity individuals *on average*. It is important to see that this is true only because high-productivity individuals have an equal chance of playing the role of either decision-maker or partner when they interact with other high-productivity individuals. If some high-productivity individuals always played the role of decision maker with other high-productivity individuals, they would be exploited all the time

by those high-productivity partners, which would drastically reduce their outside options when bargaining with low-productivity individuals, preventing the evolution of proportionality. Thus, in our model the evolution of proportionality depends as much on the possibility of changing *roles* as on the possibility of changing *partners*. In real life, this is the equivalent of having a rich and varied social life with multiple cooperative opportunities in which one is not always in the worse bargaining position [3, 4].

3.2 Theoretical problems with partner choice

Partner choice is an intrinsically complicated subject. The existence of a wide variety of cooperative partners to choose from means that a wide variety of social strategies can coexist and provide the same benefits, complicating evolutionary analysis. For example, an individual's acceptance of low rewards as a decision maker could be compensated by the low rewards she herself makes as a partner. Or some low payoffs received when interacting with low-productivity individuals could be compensated by high payoffs received when interacting with high-productivity individuals.

These effects explain why a quick look at the evolved strategies of individuals is not always enough to find a pattern of proportionality. This is especially true with neural networks working on a continuum of productivities or effort. While, as we have shown, the theoretical fitness-maximizing behavior is to offer an amount proportional to one's own relative contribution, it is not necessarily the case that neural networks will produce proportional offers for the *whole* range of inputs they are exposed to. Imagine an individual who offers proportional rewards only to the best producers in the population, while offering less-than-proportional rewards to other individuals. At the evolutionary equilibrium, our model predicts that these unfair rewards will be rejected. But as long as finding a new partner is not costly, being rejected does not lead to a loss of fitness. As a consequence, any individual can offer less-than-proportional rewards to a

fraction of the population, as long as another fraction still accepts the rewards she makes that are proportional. In other words, individuals can specialize in offering proportional rewards to only a fraction of the range of productivities in the population, and stop interacting with the remaining fraction. Because they stop interacting, the rewards offered to this fraction become subject to drift.

Because of this mechanism, it is possible that averaging the output of different evolved neural networks does not reveal a pattern of proportionality. In our simulations, averaging the output of 15,000 neural networks producing MARs yielded an almost perfect proportional relationship between contributions and MARs (main paper, Fig. 3C). Plotting the average output of 15,000 neural networks producing *rewards* did not show such a perfectly proportional relationship, although it was not far from it. Here, it is important to remember that despite this variability in the rewards that are extended, proportionality prevails when we look only at the interactions that *actually take place*: only proportional rewards are accepted at the evolutionary equilibrium, as evidenced in Fig. 3B of the main article.

Finally, problems of neutrality add complexity to the analysis. Although at the beginning of our simulations raising MARs drove the evolution of proportional rewards, once proportional rewards had spread in the population, the selection pressure to maintain high MARs disappeared: if all individuals offer rewards of r, requesting r or $r - \epsilon$ as a decision maker brings the same payoff. Because of drift, MARs can thus start to decrease, and in turn partners will be selected to decrease their rewards to try to exploit those undemanding decision makers. This exploitation cannot last for long, as it soon revives the selection pressure to increase MARs, but the dynamic exists. Although it is rather easy to conceptualize why, under appropriate conditions, partner choice leads to proportionality, the actual dynamics underlying this result are far from straightforward to understand.

References

[1] Jean-Baptiste André and Nicolas Baumard. Social opportunities and the evolution of fairness. *Journal of theoretical biology*, 289:128–35, nov 2011. doi: 10.1016/j.jtbi.2011.07.031.

[2] JM McNamara, Zoltan Barta, Lutz Fromhage, and AI Houston. The coevolution of choosiness and cooperation. *Nature*, 451(7175):189–192, jan 2008. doi: 10.1038/nature06455.

[3] Polly Wiessner. Leveling the hunter: constraints on the status quest in foraging societies. In *Food and the Status Quest: An Interdisciplinary Perspective*, pages 171–192. Berghahn Books, Oxford, UK, p. wiessne edition, 1996.

[4] Hillard S Kaplan, Paul L Hooper, and Michael Gurven. The evolutionary and ecological roots of human social organization. *Philosophical transactions of the Royal Society B.*, 364 (1533):3289–99, nov 2009. doi: 10.1098/rstb.2009.0115.
